# Splenic Rupture and Malignant Mediterranean Spotted Fever

**DOI:** 10.3201/eid1406.071295

**Published:** 2008-06

**Authors:** Laura Schmulewitz, Kaoutar Moumile, Natacha Patey-Mariaud de Serre, Sylvain Poirée, Edith Gouin, Frédéric Mechaï, Véronique Cocard, Marie-France Mamzer-Bruneel, Eric Abachin, Patrick Berche, Olivier Lortholary, Marc Lecuit

**Affiliations:** * Université Paris Descartes, Hôpital Necker-Enfants Malades, Paris, France; † Institut Pasteur, Paris, France

**Keywords:** Spleen, rupture, malignant, spotted fever, letter

**To the Editor:** Mediterranean spotted fever (MSF) is a *Rickettsia conorii* infection endemic to the Mediterranean. In this case, a 55-year-old man was referred to the Necker-Enfants Malades Hospital, Paris, France, for fever, myalgia, and hypotensive shock. The patient had been in Southern France (Montpellier) 6 days before symptom onset and had been bitten by a tick on the left hand. Four days later, he reported fatigue, fever (39°C), and myalgia. His medical history showed polycystic kidney disease, which had necessitated hemodialysis and a kidney transplant. He was receiving ongoing treatment with an immunosuppressive regime of cyclosporine, prednisolone, and tacrolimus; his baseline hemoglobin level was 15 g/dL, and creatinine level was 230 μmol/L.

At admission, the patient’s temperature was 39.5°C, blood pressure 55/40 mm Hg, and heart rate 104 beats/min. Physical examination showed a diffusely tender abdomen with guarding, no hepatosplenomegaly, a nontender renal transplant, and no lymphadenopathy. Results of cardiovascular, respiratory, and neurologic examinations were unremarkable. A diffuse maculopapular cutaneous eruption was noted on the lower limbs; no eschar was detected.

Laboratory analyses showed the following values: hemoglobin 7.9 g/dL, platelet count 115 × 10^9^/L, leukocyte count 6.7 × 10^9^/L (neutrophils 5.2 × 10^9^/L, lymphocytes 1.4 × 10^9^/L); serum creatinine 466 μmol/L, and C-reactive protein 156 mg/L. Blood cultures were negative. Serologic study results were negative for HIV, hepatitis viruses, Epstein-Barr virus, cytomegalovirus, *Legionella*, *Mycoplasma*, *Coxiella*, *Bartonella*, *Leishmania*, and *Toxoplasma* spp. Serologic testing obtained at day 1 was negative for spotted fever group (SFG) rickettsiosis.

A computed tomographic scan showed hemoperitoneum secondary to a ruptured subcapsular splenic hematoma (Appendix Figure), and an emergency splenectomy was performed. Histopathologic evaluation of the spleen showed white pulp atrophy; the red pulp indicated congestion and ill-defined nodules, varying in size and comprising macrophages, polymorphonuclear neutrophils, and necrotic cells (Figure, panels** A**, **B**). Skin biopsy of the macular eruption on day 2 demonstrated a leukocytoclastic vasculitis with nonocclusive luminal thrombi in the dermal capillaries (Figure, panel** C**).

**Figure:**
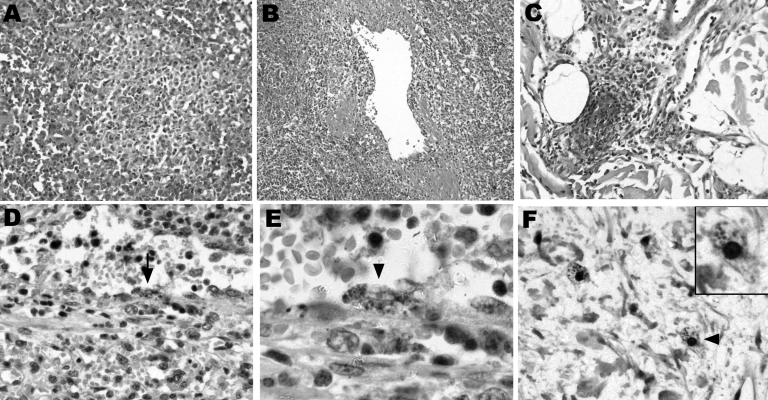
Histopathologic and immunohistochemical labelings of spleen and skin tissue samples. Tissue samples were fixed in 10% formalin, paraffin-embedded, and examined after hematoxylin-eosin staining, Gimenez staining, or immunostaining with the R47 anti-*Rickettsia conorii* polyclonal rabbit antibody. The spleen red pulp indicated congestion and ill-defined nodules varying in size and comprising macrophages, polymorphonuclear neutrophils, and necrotic cells (A, magnification ×100). A diffuse macrophage infiltration with abundant hemophagocytosis (not shown) and venulitis (B, magnification ×50) was also observed. In the skin, leukocytoclastic vasculitis with focal vascular necrosis and nonocclusive luminal thrombi were noted in dermal capillaries (C, magnification ×100). Intracellular images evocative of rickettsiae were observed in the splenic arteriolar endothelium upon immunohistochemical staining (D, arrow, magnification ×200; magnified view shown in E, arrowhead, magnification ×500). No infected cells were observed in nodular inflammatory splenic lesions. Immunohistochemical staining also disclosed intracellular immunolabeled dots in cells that could correspond to infected dermal macrophages (F, arrowhead, magnification ×300; magnified view shown in inset, magnification ×600), at a distance from the vascular alterations. Endothelial cells of dermal capillaries were also immunolabeled (Appendix Figure).

Universal 16S rRNA gene PCR amplification on spleen and skin tissue samples and direct sequencing identified an *R. conorii*–specific 16S rRNA sequence match. We confirmed this by using primers for *gltA* and *ompA* specific for *R. conorii*. Immunohistochemical staining demonstrated *Rickettsia* in endothelial cells and macrophages in the spleen and skin (Figure, panels** D**–**F**). Blood culture, skin biopsy specimens, and splenic tissue cultures were subsequently *R. conorii* positive. Doxycycline therapy (100 mg intravenously twice a day) was instituted at day 2 because rickettsiosis was suspected. The patient dramatically improved within 72 hours and remained well 36 months after diagnosis.

MSF is a rickettsiosis belonging to the tick-borne SFG caused by *R. conorii*, an obligate intracellular bacteria transmitted by the dog tick *Rhipicephalus sanguineus.* Endemic to Mediterranean countries, MSF generally results in a benign febrile illness accompanied by a maculopapular rash, myalgia, and local black eschar at a tick bite inoculation site. A minority of persons seeking treatment display a malignant form, which results from disseminated vasculitis associated with increased vascular permeability, thrombus-mediated vascular occlusion, and visceral perivascular lymphohistiocytic infiltrates (*1*). Focal thrombi have been identified in almost all organs of patients with fatal cases. Manifestations of MSF include neurologic involvement, multi-organ failure, gastric hemorrhage, and acute respiratory distress syndrome; the case-fatality rate is 1.4%–5.6%.

Splenic rupture has been reported in the course of infection with several microbial agents, including Epstein-Barr virus (*2*), HIV, rubella virus, *Bartonella* spp. (*3*), *Salmonella* spp., mycobacteria (*4*), and *Plasmodium* spp. (*5*). Splenomegaly as a result of MSF has also been documented previously (*6*); however, splenic rupture in the context of tick-borne illness has only previously been reported for *R. typhi* (*7*) and *Coxiella burnetii* infections (*8*).

SFG rickettsioses have rarely been described in transplant recipients. Barrio et al. reported a case of MSF in a liver transplant recipient with clinical resolution of infection (*9*), and a case of Rocky Mountain spotted fever after heart transplantation has been described (*10*).

Seroconversion remains the principal diagnostic tool for the rickettsioses, but often no detectable antibody is found in the early phase of the disease. Spleen and skin tissue samples allowed rapid 16S rRNA gene PCR and sequencing before the results of other diagnostic procedures were obtained. Immunostaining allowed detection of *R. conorii* in spleen and skin tissue samples and illustrated the cell tropism of this intracellular bacterium for cells morphologically similar to endothelial cells and possibly macrophages. Although *R. conorii* infection of postmortem human splenic samples from patients with fatal cases has been documented by immunohistochemical testing, *R. conorii* has not been described previously in spleen tissue of those who have survived malignant MSF.

This case expands the spectrum of infectious agents associated with spontaneous splenic rupture and solid organ transplantation. Rickettsioses are a significant risk both for those living in disease-endemic regions and for international travelers. To facilitate early detection and treatment, physicians must be vigilant for atypical symptoms, especially in immunocompromised persons.

## Supplementary Material

Appendix Figure Coronal view of unenhanced abdominal computed tomography demonstrating splenic enlargement with endocapsular hematoma and intraperitoneal hemorrhage (arrows).
