# Supplementary figures and images for: Splenic Rupture and Malignant Mediterranean Spotted Fever
Source: Emerg Infect Dis. 2008 Jun;14(6):995–7. doi: 10.3201/eid1406.071295 (PMC2600289; doi:10.3201/eid1406.071295)

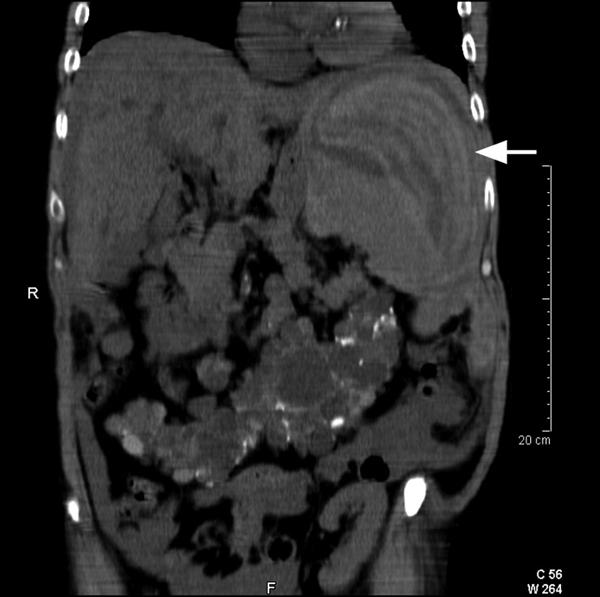

Supplement: Appendix Figure — Coronal view of unenhanced abdominal computed tomography demonstrating splenic enlargement with endocapsular hematoma and intraperitoneal hemorrhage (arrows). [file 07-1295_app-s1.gif]
